# What do we need to consider when planning, implementing and researching the use of alternatives to face-to-face consultations in primary healthcare?

**DOI:** 10.1177/2055207616675559

**Published:** 2016-11-21

**Authors:** Helen Atherton, Sue Ziebland

**Affiliations:** 1 Division of Health Sciences, Warwick Medical School, University of Warwick, Coventry, UK; 2 Nuffield Department of Primary Care Health Sciences, University of Oxford Radcliffe Observatory Quarter, Oxford, UK

**Keywords:** Primary healthcare, remote consultation, communication, policy

## Abstract

**Objectives:**

Communications technologies are variably utilised in healthcare. Policymakers globally have espoused the potential benefits of alternatives to face-to-face consultations, but research is in its infancy. The aim of this essay is to provide thinking tools for policymakers, practitioners and researchers who are involved in planning, implementing and evaluating alternative forms of consultation in primary care.

**Methods:**

We draw on preparations for a focussed ethnographic study being conducted in eight general practice settings in the UK, knowledge of the literature, qualitative social science and Cochrane reviews. In this essay we consider different types of patients, and also reflect on how the work, practice and professional identities of different members of staff in primary care might be affected.

**Results:**

Elements of practice are inevitably lost when consultations are no longer face-to-face, and we know little about the impact on core aspects of the primary care relationship. Resistance to change is normal and concerns about the introduction of alternative methods of consultation are often expressed using proxy reasons; for example, concerns about patient safety. Any planning or research in the field of new technologies should be attuned to the potential for unintended consequences.

**Conclusions:**

Implementation of alternatives to the face-to-face consultation is more likely to succeed if approached as co-designed initiatives that start with the least controversial and most promising changes for the practice. Researchers and evaluators should explore actual experiences of the different consultation types amongst patients and the primary care team rather than hypothetical perspectives.

## Background

Communications technologies are routinely used by the public in everyday life, and there is an expectation that this should extend to healthcare. Globally, the use of such technologies in healthcare is variable. In Denmark, the option to use email for consultations in general practice became mandatory in 2009 – a measure intended to raise the quality of services delivered to patients. In 2013 there were four million consultations conducted this way.^1,2^ The US has well-established options, including patient portals for online access to clinicians and routine telephone consultations, which are offered by several of the large health maintenance organisations, under fee-for-service arrangements.^3^ In Finland, emails between doctors and patients have been an unremarkable part of care for over a decade.^4^ Mobile devices in parts of Africa have vastly increased access to the telephone, as well as the Internet; the potential impact is arguably more transformational than countries with pre-existing landline networks.^5^ In the UK, there has already been much investment in the use of telephone and website services to provide patients with a ‘non-emergency’ point of entry to the healthcare system,^6^ but this use has not extended to traditional care settings. Policymakers have suggested that alternatives to face-to-face consultations in the general practice setting could have a transformative (and positive) impact through alleviating staff workload and improving patient access, but their use is far from routine.^7,8^

Research on these alternative methods of consultation is in its infancy. The Cochrane reviews of the evidence about the use of email^9^ and telephone^10^ consultations found equivocal evidence from trials that are rarely high quality. In countries where alternative forms of consultation are on the agenda, primary care professionals have responded with both readiness and reluctance (but rather more of the latter). Opinion pieces and policy documents also reflect a mix of enthusiasm for innovation and resistance to change.^11,12^ Patients, when they have been asked, are usually in favour of consultation options which appear more convenient and efficient.^13^

This essay arises from our preparations for a focussed ethnographic study, which we are currently conducting in eight general practice settings in the UK.^14^ We draw on our knowledge of the literature and our experience in primary care research and practice, qualitative social science and Cochrane reviews, as well as formal and informal discussions with patients and general practitioners (GPs), and responses from conference audiences. We believe that this approach, which supplements our own contributions to Cochrane reviews of the evidence, is appropriate to the task in a rapidly moving field.

Recent years have seen a plethora of small and local pilot projects and commercial initiatives around specific systems,^15,16^ which proliferate in an environment of patchy and inconclusive evidence. Because these models are being promoted as potentially cost-effective solutions in primary healthcare, their growth will likely continue unless major safety and economic consequences become apparent. We suggest that some cool reflection is needed about what should be considered when planning, implementing and researching alternatives to the face-to-face consultation. Our aim in this essay is not to synthesise or review the published evidence, but to consider, more widely, what might be the effects of alternative forms of consultation at the level of the patient, the organisation and the professions, and the implications for the consultation, the practice environment and broader health system. We discuss what might be gained, and what lost, through using alternative forms of consultation. We consider different types of patients, for example people whose circumstances that make it hard to visit the practice, and also reflect on how the work, practice and professional identities of different members of staff in primary care might be affected. We aim to provide some thinking tools for policymakers, practitioners and researchers who are involved in planning, implementing and evaluating alternative forms of consultation in primary care.

In structuring our essay, we have drawn on the sociological framework of healthcare work and organisation for information and communications technology (ICT) initiatives as outlined by Halford et al.^17^ Their theoretical approach views that the introduction of new ICT applications threatens to disrupt healthcare work and organisation by disrupting social orders mediated by inter-relations of power, knowledge and identity. The analytical framework positions ICT initiatives within orderings of healthcare work and organisation, with ICT applications posing three potential disruptions to the organisational, professional and spatial dimensions of healthcare work and organisation. We chose this framework because it helps us to understand, at a general level, how ICT initiatives disrupt the prevailing order of healthcare.

We use these dimensions to structure our discussion, with the acknowledgement that these are inter-related and ongoing processes that are shaped by healthcare work and organisation, and are not occurring independently.

Our starting point in compiling this essay was the recognition that any new technology can be highly disruptive to practice, even if it ultimately benefits both the service and the practice population. We also recognise that resistance to change is normal,^18^ especially if staff are under pressure or feel that the innovation may interfere with the most cherished aspects of their role.

**Table 1.** What to consider when planning, implementing and researching alternatives to face-to-face consultations.

	Key questions
*Organisational disruptions and dynamics*	
Patient awareness	How could patients find out what methods of consultation are offered by their doctor?
Organisation of alternatives within the healthcare setting	How will alternatives be scheduled into existing practice? What impact will alternatives have on reception and administrative staff work patterns? What are the agreed rules of engagement for use of alternatives? What contingency is in place to ensure that communication by asynchronous alternatives is responded to, and in a timely fashion? How will the expectations of all parties be managed? How can consultations be appropriately administered to avoid duplication of effort? How will alternative forms of consultation be documented in the medical record, especially when consulting remotely from the practice? Is reimbursement for alternative consultations appropriate? What are the arrangements for reimbursement?
Ensuring safety when using alternatives in the healthcare setting	What are the potential patient safety issues associated with using alternatives? How are (or might) these be mitigated? Are there risks for patient privacy and confidentiality?
Organisation of space in the healthcare setting	What are the contingency arrangements for technology failure?
*Professional disruptions and dynamics*	
Interface between technology and individual practice	What did the designers intend it to do – and (more important) how is it used in practice?
Proximity of professional to patient	Does it allow eye to eye contact? Is it real time or asynchronous? What is lost in comparison with the co-present consultation? What is the effect on valued aspects of primary care such as the relationship and continuity of care? What is the alternative appropriate for? Is the alternative offering a replacement for the face-to-face consultation or is it complementary? Is there a risk of misunderstanding due to the change in medium and can this be mitigated for? Will alternatives change how patients communicate?
Primary care teams	How are the roles of different team members affected by use of alternatives? Are there implications for staffing in the practice?
Professional indemnity	How is medico-legal protection in relation to non-face-to-face consultations organised and understood in the practice setting?
Healthcare professional attitudes	What are the views and concerns of different members of the team about alternatives to the face-to-face consultation?
Healthcare professional skills	What skills are needed? Is training and support available? Will patients require training, or guidance in using alternatives?
Broader changes to professional practice	Will the introduction of alternatives allow for flexible working? If so, might this impact on primary care staffing: recruitment and retention? Are there cost implications?
*Spatial disruption and dynamics*	
The nature of the communication medium	Who was involved in setting up the system and whose work was considered? Is the rationale for introducing alternatives clear and understandable to all staff members? What impact does it have on all of the different members of the team? Whose core values and interest are served? How is resistance enacted, and by whom?
Patient interface with alternatives to the face-to-face consultation	How will patient experiences of using alternatives be collected and recorded? Are there types of consultation that are preferred face-to-face? What about patients from groups who are often assumed to be disadvantaged in relation to alternative methods (older, disabled, less educated, language difficulties)? How might patients use the opportunity to share digital files with their doctors?
*Unintended consequences*	Are there consequences (either positive or negative) for other elements of the practice, or other aspects of care provision? Consequences for other parts of the health system (use of emergency helplines, hospital emergency departments, etc.)? Do (how do) staff and patients modify new forms of consultation to better meet their needs? How else might the planner, implementer or researcher identify unintended consequences?

## Organisational disruptions and dynamics

New initiatives disrupt existing organisational practices. This is generally assumed, by those initiating them, to occur in a positive fashion, bringing benefit.^17^ We suggest exploring this notion in relation to patient awareness, organisation of alternatives within the healthcare setting, ensuring safety when using alternatives in the healthcare setting and organisation of space in the healthcare setting (Table 1).

### Patient awareness

In settings where the availability of alternative forms of consultation is a matter for individual practices, rather than required by policy directives, then it is worth asking how patients find out what is available to them at their practice. In a survey of patients’ reasons for not consulting their doctor by email, the lack of awareness of the possibility of an e-consultation was one of the main reasons for non-use.^19^ We should observe whether there is a poster in the waiting room. Is there a reminder at the reception desk? Could patients find this information in the practice leaflet, or on the practice website? Indeed, does the practice have a website? Is there a patient portal inviting email enquiries? Does the receptionist booking the appointment suggest an alternative form of consultation to patients (and if so, which patients)? Or do staff select ‘sensible’ patients’ for email consultation? Primary care professionals have described selectively offering alternatives to patients they feel are able to use them appropriately.^20,21^ The answers to these questions will clearly affect uptake and the attitudes both of patients and members of the primary care team.

### Organisation of alternatives within the healthcare setting

Several of the misgivings that have been raised about alternative consultations relate to the organisation and administration of the practice rather than the consultation itself. Common concerns include: What will happen if a part-time member of staff doesn’t pick up an urgent email? Will alternative methods introduce inefficiencies for the practice?^13^ Staff sometimes express concerns about whether patients will exercise their options responsibly, fearing that the relative ease of sending an email (or a stream of emails) may mean that some patients will over-consult or misrepresent their symptoms.^22,23^ While evidence is limited, in settings where email consultations have been introduced they have not, as yet, opened the floodgates for patient demand.^24^ Even in practices and health systems where patients have had the right to email their family doctors for some time, these alternatives are not widely used.^25,26^ In Denmark, where email consultations are a standard part of primary care, some doctors admit to managing their patients expectations by deliberately delaying their responses to non-urgent emails.^27^

Potential inefficiencies include duplicated consultations if patients consult remotely and then attend the practice or need a home visit.^28–30^ A study of telephone triage in general practice found that where telephone triage led to a face-to-face consultation, the duration of this subsequent face-to-face consultation was no shorter despite a clinician speaking with the patient during the telephone encounter.^31^

Primary care is set up to deliver the face-to-face consultation. As yet, there is little evidence about how best to time, conduct and record other forms of consultation.^22,32,33^ These uncertainties make changes to service delivery difficult.^9,34^ Alternatives to face-to-face consultations could be managed in scheduled daily or weekly sessions or (systems permitting) in between other clinical appointments, in transit, or from home. An email, and its attachments, may be transferred into the patient record with greater efficiency than notes from a consultation, but if the primary care professional is replying to emails away from their office it is easy to envisage problems occurring in record-keeping.

Arrangements for recognising and reimbursing some of these alternatives remains something of a work in progress.^35,36^ For example, Danish GPs have reported a lack of consensus about when emails are more akin to ‘social exchanges’ of pleasantries than consultations.^37^ In the US, problems have arisen where reimbursement for Medicare patients is at the discretion of individual insurers, with many patients not reimbursed for alternative types of communication with their healthcare provider.^38^

Different alternatives also differ in their impact on practice organisation. The face-to-face consultation is usually booked via reception staff. This is also the case for most telephone consultations. Email can allow patients to bypass the gatekeeping role of the reception staff and obtain direct contact with the primary care professional, or whoever is allocated the task of replying to the email.^21,39^ This prospect is sometimes viewed as unacceptably disruptive by physicians,^40^ although, as we have discussed above, patients tend to like the improved access.

### Ensuring safety when using alternatives in the healthcare setting

Patient safety is crucial in any form of consultation, but alternatives present an unknown in terms of what these issues might be. Despite patient safety being cited as a reason to be wary of introducing alternatives,^41^ there is very little documentation of what these might involve. Patient privacy and confidentiality are described as important, but reports of privacy and confidentiality breaches are few and collection of these data uncommon. The Cochrane review of trials relating to email for consultation found that the trials did not report any harms; but this is not, of course, the same as stating with confidence that no harms occurred.^9^ There is much work to be done in identifying potential patient safety issues and mitigating the risk associated with these.

As well as considering risk in medico-legal terms, consideration must be made for the medico-legal support available to primary care teams. Medical indemnity fees are already a significant expense for primary care practices.^42^ Doctors seeking advice from medico-legal organisations are likely to receive conflicting advice and, in some cases, following their enquiry, may see an increase in their annual fees. This situation has implications for the introduction of alternatives to the face-to-face consultation and some teams may conclude that additional costs will likely outweigh any efficiency savings.

### Organisation of space in the healthcare setting

To benefit from video conferencing, practices may need to allocate a well-lit, private area for the staff to use and reliable connections so that screens do not freeze mid-consultation.^40,43^ The same, of course, applies to the systems that the patients are using.^44^ Reliable contingency arrangement may be needed in case of technological failure. The potential for ‘freezing’ or image breakdown during a video consultation may have clinical consequences – for example, it can be particularly disturbing for people with mental illness.^43^

## Professional disruptions and dynamics

Professional identities and roles are important. New initiatives have the potential to disrupt professional knowledge and practice, and inter-professional relations.^17^ We suggest exploring this notion in relation to the interface between technology and individual practice, proximity of professional to patient, primary care teams, professional indemnity, healthcare professional attitudes, healthcare professional skills and broader changes to professional practice (Table 1).

### Interface between technology and individual practice

As the sociology of science and technology has demonstrated repeatedly, what a technology *is* cannot be determined by its design but by how it is used.^45^ The potential of a new technology is ‘not pre-given but is shaped in practice’;^46^ In studying these alternative consultation technologies we need to place at the forefront of the analytic agenda how the technology is used (and resisted).^47^ This will often require direct observations of the work practices, since the actors involved may not be aware – or may find it difficult to report – when and how their interactions are shaped by the technology.

The complex relationship between the characteristics of the technology itself and the way that people use, avoid and adapt it in everyday practice also means that it may be hard to wrestle transferable outcomes from the field. It may be difficult for users to separate what are truly technological issues concerning hardware and software, and what arises as a result of introducing new practices. But a good start will be to explore the practice around the technology rather than assuming that its effects are constant (for here lies a sure route to non-transferable interventions).

### Proximity of professional to patient

The common feature of all alternatives to the face-to-face consultation is, of course, that they are not face-to-face; therefore, it is inevitable that they will be compared with this ‘ideal’ form of consultation.^48^ Physical co-presence within the consultation has been a taken-for-granted characteristic^49^ and is a central component of the patient–doctor relationship within primary care.^50,51^

The healthcare professional’s identity is also tied up in the face-to-face consultation, whether conducted in their own office or in the patient’s home. This is where professionals demonstrate their clinical knowledge and skills and make decisions about the meaning of the patient’s sensations and symptoms.^52^ The consultation is also where the doctor performs care and (ideally) develops the mutually trusting relationship and continuity of care that underpins a highly valued aspect of primary care.^53^ A different medium inevitably changes some aspects of the performance of the consultation; these elements are either lost or may need to be expressed in a different way or performed at a different time, for core elements of the doctor–patient relationship to be maintained.^54,55^

Primary care consultations typically include history taking, physical examination and investigation.^51,56,57^ There is particular uncertainty around the ‘rules of engagement’ for email and video consultations.^21,27,58^ The proximity with the patient that is afforded in the traditional face-to-face consultation permits diagnostic cues such as smelling the patients skin and breath, noting how they walk into the room and using casual contact, such as shaking hands, to assess skin temperature and tone.^59,60^ The professional may lose some of their ability to check the patients understanding, which is often conveyed via non-verbal communication.^61,62^ As yet, there is little research indicating whether misunderstandings are increased or diminished with alternatives forms of consultation.

Doctors also describe ‘door handle’ issues, whereby patients divulge new information just as they are about the leave the consultation. Conversely, remote (and especially asynchronous) exchanges may offer other opportunities to reflect on the consultation and seek second opinions, which could improve care (and avoid loss of professional face).

Some primary care consultations do not require the use of honed clinical skills; routine clinical issues may be more efficiently dealt with without direct contact. Blumenthal, writing in 2010 in the context of US healthcare, anticipated a future primary care workplace with a dramatic change to workflow through the online management of administrative issues such as prescription renewals, referrals, appointments, third-party authorisations and paperwork.^63^ He also envisaged that before the consultation, patients would be routinely asked to complete an online questionnaire (about the problem, symptoms, recent changes). A self-confessed optimist about the impact of the Internet on health and care, he anticipated that patients who would want to be full partners in their care would have access to their entire health records. Thus, ‘Everything providers know about patients, and everything they do with and for them, will be mediated by software. The computer will be as omnipresent and important as the stethoscope.’ p14.

### Primary care teams

Very little is known about the implementation of alternative forms of consultation in primary care, but there is a wealth of literature which supports the need for new systems to take account of the values and practices of the team.^64–66^ Implementation of new approaches to the primary care consultation is particularly unlikely to be successful if the technology does not fit into the work patterns of front-line members of the practice team.^67^ The impact of alternative forms of consultation on collaborative working, division of labour, continuity and multidisciplinary care have received minimal attention to date.

At a minimum, primary care teams include doctors, nurses, allied health professionals, practice managers, administrators and reception staff. New technologies are unlikely to be accepted by staff who doubt that the technology will help them to fulfil their core roles, which evidently differ. In the literature, doctors’ views feature most prominently and the perspectives of administrative staff and reception staff are very rarely considered. This may seem surprising given the centrality of the administrative staff and receptionists in administering and allocating appointments. As the usual point of contact for patients seeking appointments with clinical staff, the receptionist is key to whether patients are guided towards any alternative method of consulting.

How the alternative is introduced and implemented in the practice will affect how the change is managed, and resisted, and with what consequences. Clinical staff in particular may be reluctant to make adjustments which they see as driven by non-clinical, political, external, administrative or financial pressures. Studies to date have rarely indicated whether the introduction of the alternative was agreed across the practice team, or introduced by a single member of the team. Given the complexity of the setting and the multiple potential effects of the alternative approaches for different parties, we suggest that these matters should be considered key in future work.

### Professional indemnity

Related to the lack of guidance or consensus on best practice, patient safety and the risk of litigation are often raised when alternative forms of consultation – or, indeed, almost any changes to practice – are proposed.^20,68^ This could be understood as a proxy reason, given that highlighting concerns about patients’ safety is undoubtedly a more ‘acceptable’ form of resistance than voicing concerns about threats to professional identity and power. Yet, there is also some evidence that clinicians’ safety concerns are translating into more cautious prescribing behaviour; for example, primary care doctors shown to be more likely to prescribe antibiotics during an e-visit than when they consult face-to-face.^69^ This may reflect uncertainty around the medico-legal consequences of this type of prescribing. When we are trying to understand how alternatives are working, we need to be alert to how safety netting procedures are enacted.

### Healthcare professional attitudes

When healthcare professionals are asked about their views on using alternatives to the face-to-face consultation, concerns tend to focus on whether their clinical duty to provide safe and effective care might be compromised.^70,71^ Much of this concern relates to the potential impact of these additional consultation methods on their workload. Fears expressed include increases in consultation volume^72,73^ and increased administrative load.^74^

Those with experience of successfully using alternatives in their own practice raise similar issues, still feeling uncertain about the long-term effects on their workload and, consequently, their patients.^21^ Research suggests that any new technology needs to be seen to enhance what the professional sees as their core role,^65^ otherwise it is unlikely to be accepted into practice.^75,76^

There have been far fewer studies collecting the views and experience of practice nurses on alternative consultation methods, but there is evidence that nurses feel their role requires proximity to the patient.^55,77,78^ Intriguingly, Tjora, in a Norwegian study of nurses working in emergency medicine, found that they were more assertive and gave more advice when consulting remotely rather than face-to-face.^79^ In a study of a telehealth self-care support system for people with chronic health problems, the nurses who were providing the service positioned their work as ‘proper nursing’ while primary care nurses whose practices were using the telecare system suggested that the calls with patients were ‘just chat’ and doubted that real nursing could be delivered via the telephone.^80^ This recalls the work of the Dutch social scientist Jeanette Pols, who has described a ‘professional fear’ among nurses that

the use of telecare systems will make it more difficult for nurses to act competently and responsibly when looking after patients, particularly because care is at a distance and the nurse is not physically present. Although videoconferencing often has a better press, monitoring devices not involving eye to eye contact are regarded with suspicion. The horror images seem to be *negligence* and *coldness*: the patient is ‘on telecare’, but gets worse, without anybody having noticed it.^81^ p375

Pols has challenged the contrasting association of ‘technology’ with coldness, distance and efficiency and ‘care’ as warm, proximal and emotionally involved, pointing out in ‘Care at a distance’^82^ that this is a false dichotomy. Her ethnographic work with elderly patients with heart failure, and the nurses and other staff involved in a home video telecare system, found that the nurses were able to perform ‘care even closer’ rather than ‘care at a distance’ through using the technology.

### Healthcare professional skills

It is important to consider whether the technology is familiar and easy for both parties to use or whether it requires new skills. Varsi et al.^83^ recommend that patients should be shown how to use a system at a point when it is relevant to them, rather than as part of a general induction to their doctor’s practice. If the information does not come at the right time the patient may not remember the system or (likely in a fast moving field) the system may have changed by the time they come to use it. Some healthcare professionals worry that their lack of confidence with technology may be exposed, and that such exposure might undermine their authority.^84,85^ In a study of breastfeeding support via video consultation, lactation consultants were concerned about technical issues such as the quality of images, yet patients were very satisfied with the remote consultation. The lactation consultants were not confident about undertaking clinical assessments via video – a need which the authors concluded could be addressed by specific training in using the medium.^44^ While the balance of power within the consultation may change if the primary care professional’s skills comes under patient scrutiny,^86^ this is not necessarily damaging and could even be a helpful shift in the balance of the longer-term relationship.

Another consideration is whether the method allows the parties involved in the consultation to add web links and attachments. Does it leave a record of the interaction that can be accessed by the participants and others? What the technology can and cannot do, how difficult it is for the participants to use, and what support is needed to help the parties to become competent in its use, are all important considerations.

### Broader changes to professional practice

Alternative forms of consultation offer a change to the location and time of work for healthcare professionals; remote methods of consulting allow for flexible working, for instance, drawing retired clinicians back into the workforce by allowing them to consult remotely, thus also addressing workforce shortages.^87^ There is also scope for part-time staff to consult remotely,^88,89^ adding flexibility to their work. For example, in Pakistan 70% of medical students are female but only 23% of practising doctors are female. A pilot study in Sultanabad, Pakistan (the DoctHERS project) has sought to address this by allowing women doctors who have left the workplace ‘due to marriage or children’ to consult patients via Skype from home, serving people who would not otherwise have access to quality healthcare.^90^

Globally, primary care professionals are under pressure in the workplace. Alternative methods of consultation have been promoted in some settings as a solution to increased demands from an aging population, managing long-term conditions.^91^ The emphasis has often been on (cost-) efficiency, which professionals may see as conflicting with their professional identity and ability to deliver high-quality patient care.^65^

Worries about using a form of consultation that is not ‘tried and tested’ tie in with concerns about the long-term sustainability of primary care and how it is funded and staffed. There has been a steady move towards involving non-medical staff – for example, physician assistants – in delivering care.^92,93^ Now widely used in the US, physician assistants are mooted as a way of addressing the primary care workforce crisis in Europe too. There is the possibility that this could focus around the use of alternative forms of consultation. Recently, a UK primary care practice has advertised for a physician assistant whose role will be to conduct consultations via alternative methods only.^94,95^

## Spatial disruption and dynamics

Spatialisations of work and organisation relate to identity and power and how they change when a new initiative is introduced. This is often seen as being a technical or economic issue, when occupying space is more complex than this, comprising the representation, meaning and practice in the work and organisation.^17^ We suggest exploring this notion in relation to the nature of the communication medium, patient interface with alternatives to the face-to-face consultation and unintended consequences (Table 1).

### The nature of the communication medium

There are already many different technologies that patients could use to consult their doctor without meeting face-to-face – for example, telephone, email, Short Message Service (SMS) and video communication. While specific platforms are likely to be superseded in a fast changing field, we can differentiate according to whether the method provides (moving) images, audio or written content, and whether the exchange is in real time or asynchronous. Asynchronicity allows the healthcare professional to draw upon external resources or check evidence, perhaps providing sources of information for the patient.^13,83^ For patients, it allows them time to construct an enquiry, perhaps with help from family or friends, and to send follow-up questions that occur after a consultation. Methods that allow video connection give participants the opportunity to view a symptom (such as a rash) and also, potentially, a view of the patient’s home setting. Video may also help establish a relationship, and enable the participants to monitor non-verbal cues and check understanding.^96^

### Patient interface with alternatives to the face-to-face consultation

Where patients have been offered alternative methods of consultation, they usually report liking them.^97,98^ Email and telephone consultations remove the need to attend the GP or nurses’ professional space, which tends to be viewed as a benefit by patients.^13,39,69^ Other reported benefits include the convenience of being able to consult whilst at work,^99^ to choose when and how to consult and the perceived advantage of avoiding the doctor’s receptionist.^27,77^ The ability to communicate with one’s doctor via email means that the patient can compose a message when something is bothering them, which may be outside of office hours. The patient (and their family) may like to exchange information and attachments relevant to health and care decisions and forward these to their health professionals. Parents can photograph, record and attach digital files with images of a child’s rash, or recordings of an infrequent cough or breathing difficulty.^27,84^ For patients preparing for a visit to hospital, or recovering at home afterwards, these methods can provide a way to keep in touch without necessitating a visit.^55^

In 2002, Muir Gray’s ‘The Resourceful Patient’^100^ recognised the potential value to patients of e-consultations for which they could prepare with ‘pre-consultation prep’ to be better able to participate in decisions. He acknowledges that

not all patients will wish to avail themselves of the responsibilities and resources … however the fundamental contract between patient and clinician in the 21^st^ century should start with the assumption that the patient is competent and responsible, providing they are given the resources to exercise that responsibility. (p.112)

Email exchanges can provide a record and, perhaps, a clearer explanation and understanding than may be absorbed face-to-face.^55^ This may be particularly advantageous to those who are less articulate or confident in person, those who wish to discuss their consultation with others and those who need help with translation.^22^ Some patients may be more willing to disclose intimate or sensitive information via an email than in person or over the phone – especially if they are at work or in a public place.^27^ For others, the reverse will be true, not least because of concerns about confidentiality in emails.

Health professionals raise concerns that older patients, disabled patients, people without literacy skills and those patients who are less educated^3,26^ may be disadvantaged through alternative forms of consultation.^70^ Interestingly, there is some evidence that, for those who have Internet access, patients who are disabled, elderly, less confident or living at some distance from the practice are often amongst those who are particularly keen to use email consultations.^101^

While there is a lot of speculation about the potential benefits and disadvantages for patients, and particular subgroups of patients, much of it has been written from the healthcare professional perspective and credible empirical evidence from patients is very limited. The perspectives and experiences of patients (and especially those from groups who are assumed to be disadvantaged through the introduction of alternative methods of consulting) clearly needs further attention when designing implementing and evaluating systems.

## Unintended consequences

Any planning or research in the field of new technologies should be attuned to the potential for unintended consequences. There are numerous examples of technologies that have been tinkered with and adapted in the field,^65^ some to the extent that their initial purpose is barely recognisable. Changes in one element of care provision can have effects on other elements of care, and on the role of other staff. An example is Winthereik and Langstrup’s (2010) study in Denmark of patient and professional behaviours in response to a new portal for pregnant women.^102^ The portal was introduced to help women with uncomplicated pregnancies to self-manage, thus (it was anticipated) freeing up resources for more complicated cases. They found that the (minority) of women who engaged with the portal enacted their active and responsible involvement at the clinic rather than at home. The use of the portal, therefore, provided both more and less than was anticipated: it reconfigured relations in a way that is likely to alter the meaning of care, but not in a manner that was likely to free up resources. The healthcare practitioners, who were supposed to be using the portal to maintain a complete and shared electronic record, were instead printing a paper record and adding their own handwritten notes. The healthcare professionals ended up doing more work than before.

## Discussion

Our intention in this essay has been to consider how alternatives to face-to-face consultation in primary care might be developed and understood, bearing in mind the needs of those who plan, implement and research these alternatives. Some concerns (e.g. confidentiality, safety and litigation) have been frequently raised in the literature,^21,103^ while others (e.g. how changes to the options open to patients may affect the work practices of doctors’ receptionists and practice managers) have received very little consideration in the literature to date. Aware of these gaps, we have sought to reach beyond the confines of a literature synthesis and instead draw on our preparation for an ethnographic study in primary care, using a structure focused on how ICT initiatives sit within healthcare. Where there is a relevant literature, we have referenced it, but we also offer informed speculation (and a few tangents) which we hope will help policymakers, primary care teams and researchers to consider the potential consequences of changes to the consultation. We have made the assumption that the ‘traditional’ method of consultation is face-to-face, but acknowledge that this is based on the status quo. As far back as the 1700s, alternatives to the face-to-face consultation were in use; Edinburgh physician Dr William Cullen famously conducted consultations with his patients via letter.^104^

We have described resistance shown by healthcare professionals to the introduction of alternative methods of consultation. This type of resistance is normal and objections are often expressed via proxy reasons, for example concerns about patient safety. However, as we have discussed, elements of practice are inevitably lost when consultations are no longer face-to-face and we know little about the impact on core aspects of the primary care relationship. The ‘real’ reasons for staff enthusiasm or resistance are always likely to be elusive, but those who wish to implement these changes are more likely to succeed if they work with the practice team and the patient population in co-designed initiatives that start with the least controversial and most promising changes for the particular practice. Researchers and evaluators would be well advised to ask different groups of patients, as well as different members of the primary care team, about their actual experiences of the different consultation formats rather than add to the largely hypothetical responses that dominate the current literature.

In this essay we take a pragmatic approach as to what we need to consider when planning, implementing and researching the use of alternatives to the face-to-face consultations in primary care. We hope in applying these that the consequences, positive and negative, of implementing alternatives to face-to-face consultation in primary care can be better understood, to the benefit of patients, primary care teams and the health system.
